# Excitatory Modulation of the preBötzinger Complex Inspiratory Rhythm Generating Network by Endogenous Hydrogen Sulfide

**DOI:** 10.3389/fphys.2017.00452

**Published:** 2017-06-30

**Authors:** Glauber S. F. da Silva, João P. J. Sabino, Vishaal Rajani, Tucaauê S. Alvares, Silvia Pagliardini, Luiz G. S. Branco, Gregory D. Funk

**Affiliations:** ^1^Department of Physiology, Faculty of Medicine and Dentistry, Women and Children's Health Research Institute, Neuroscience and Mental Health Institute, University of AlbertaEdmonton, AB, Canada; ^2^Department of Morphology and Animal Physiology, Sao Paulo State UniversityJaboticabal, Brazil; ^3^Department of Biophysics and Physiology, Federal University of PiauiTeresina, Brazil; ^4^Department of Physiology, Faculty of Dentistry of Ribeirao Preto, University of Sao PauloRibeirao Preto, Brazil

**Keywords:** control of breathing, hypoxia, H_2_S, cystathionine-β-synthase, AOAA, preBötzinger Complex

## Abstract

Hydrogen Sulfide (H_2_S) is one of three gasotransmitters that modulate excitability in the CNS. Global application of H_2_S donors or inhibitors of H_2_S synthesis to the respiratory network has suggested that inspiratory rhythm is modulated by exogenous and endogenous H_2_S. However, effects have been variable, which may reflect that the RTN/pFRG (retrotrapezoid nucleus, parafacial respiratory group) and the preBötzinger Complex (preBötC, critical for inspiratory rhythm generation) are differentially modulated by exogenous H_2_S. Importantly, site-specific modulation of respiratory nuclei by H_2_S means that targeted, rather than global, manipulation of respiratory nuclei is required to understand the role of H_2_S signaling in respiratory control. Thus, our aim was to test whether endogenous H_2_S, which is produced by cystathionine-β-synthase (CBS) in the CNS, acts specifically within the preBötC to modulate inspiratory activity under basal (*in vitro*/*in vivo*) and hypoxic conditions (*in vivo*). Inhibition of endogenous H_2_S production by bath application of the CBS inhibitor, aminooxyacetic acid (AOAA, 0.1–1.0 mM) to rhythmic brainstem spinal cord (BSSC) and medullary slice preparations from newborn rats, or local application of AOAA into the preBötC (slices only) caused a dose-dependent decrease in burst frequency. Unilateral injection of AOAA into the preBötC of anesthetized, paralyzed adult rats decreased basal inspiratory burst frequency, amplitude and ventilatory output. AOAA *in vivo* did not affect the initial hypoxia-induced (10% O_2_, 5 min) increase in ventilatory output, but enhanced the secondary hypoxic respiratory depression. These data suggest that the preBötC inspiratory network receives tonic excitatory modulation from the CBS-H_2_S system, and that endogenous H_2_S attenuates the secondary hypoxic respiratory depression.

## Introduction

Hydrogen sulfide (H_2_S) is a gasotransmitter that modulates neuronal excitability and synaptic transmission in the peripheral and central nervous systems (Kimura, 2014). It is produced by three main enzymes: Cystathionine γ-lyase (CSE) predominates in peripheral tissues, while cystathionine β-synthase (CBS) and 3-mercaptopyruvate sulfurtransferase (3MST) are the major contributors in the CNS (Abe and Kimura, 1996; Yang et al., 2008; Kimura, 2014). Environmental H_2_S is a long-recognized human toxin that increases breathing frequency at moderate concentrations. High concentrations decrease frequency and are also associated with respiratory and cardiac irregularities and coma (Beauchamp et al., 1984; Reiffenstein et al., 1992). High doses inhibit the activity of inspiratory networks isolated *in vitro* (Greer et al., 1995) and cause apnea and death within minutes. However, the lethal actions of exogenous H_2_S are not due to its direct inhibition of the central respiratory network because the brainstem inspiratory network, when isolated *in vitro*, continues to generate rhythm when exposed to levels of H_2_S that are lethal *in vivo* (Greer et al., 1995).

The brainstem respiratory network is sensitive to exogenous H_2_S, since application of donors *in vitro* and *in vivo* evoke a biphasic ventilatory response comprising an initial inhibition followed by excitation (Hu et al., 2008; Chen et al., 2013a,b; Li et al., 2014), or an excitation alone (Pan et al., 2011; Chen et al., 2013b). Application of exogenous cysteine (CYS, a metabolic precursor of H_2_S) to thick brainstem slices *in vitro* (Hu et al., 2008; Pan et al., 2010, 2011) or *in vivo* (Li et al., 2014) evokes the same range of responses, indicating that the network can be modulated by endogenously generated H_2_S. These data are not, however, evidence of physiological modulation. The only evidence of a physiological role for H_2_S signaling in respiratory control is the inhibition of ventilation following inhibition of H_2_S synthesis throughout the brainstem *in vitro* and *in vivo* (Hu et al., 2008; Li et al., 2014), but not all studies support a role for endogenous H_2_S in baseline respiratory activity (Pan et al., 2011; da Silva et al., 2014; Li et al., 2016). Reduction of the secondary hypoxic respiratory depression *in vitro* and *in vivo* (Pan et al., 2010, 2011; Li et al., 2016) by global application of H_2_S donors and CYS suggests that H_2_S contributes to the hypoxic ventilatory response, but evidence of a physiological role is not definitive because the H_2_S in these studies was either exogenous (i.e., when donors are applied) or derived from an exogenous precursor (i.e., when CYS is applied).

A factor that may impede detection of endogenous respiratory network modulation by H_2_S is the potential that components of the respiratory network are differentially sensitive to H_2_S (Chen et al., 2013a,b). The majority of studies exploring H_2_S signaling in respiratory control have applied H_2_S-active agents in a manner in which they affect the entire brainstem network. Simultaneous activation of excitatory and inhibitory regions by global activation of H_2_S signaling may obscure endogenous actions. Similarly, variability in the activation of the excitatory and inhibitory mechanisms or the rostro-caudal boundaries of rhythmically-active *in vitro* preparations could contribute to the variability in the reported effects of H_2_S on breathing (Chen et al., 2013a,b). The aims of this study were two-fold. First, we tested using *in vitro* and *in vivo* approaches the hypotheses that endogenous H_2_S signaling specifically in the preBötzinger Complex (preBötC), a critical site for inspiratory rhythm generation (Smith et al., 1991; Gray et al., 2001; McKay et al., 2005; Tan et al., 2008), is a source of tonic excitatory modulation under baseline conditions. Second, because H_2_S modulates signaling within other components of the afferent circuit that underlies the ventilatory response to hypoxia, namely the carotid body (Peng et al., 2010) and nucleus tractus solitarius (Austgen et al., 2011), we tested the hypothesis that H_2_S signaling in the preBötC helps shape the dynamics of the hypoxic ventilatory response (HVR). Inhibition of CBS-mediated, endogenous H_2_S production (Abe and Kimura, 1996; Asimakopoulou et al., 2013) via bath and local application of aminooxyacetic acid (AOAA) *in vitro* and *in vivo*, suggests that endogenous H_2_S provides tonic, excitatory modulation of the preBötC inspiratory network under baseline conditions and attenuates the secondary depression of ventilation that occurs during hypoxia.

## Materials and methods

All experiments were conducted in accordance with the guidelines of the Canadian Council on Animal Care and were approved by the University of Alberta Animal Ethics Committee (Protocols AUP255 and AUP256). The *in vitro* experiments were carried out using neonatal Sprague–Dawley (SD) rats (0–4) days old. The *in vivo* experiments were performed using adult SD rats (250–350 g). Rats were provided with food and water *ad libitum* and kept on a 12:12 h dark-light schedule.

### *In vitro* preparations

The neonatal rat brainstem–spinal cord (BSSC) preparation was produced as described in detail previously (Suzue, 1984; Smith and Feldman, 1987; Alvares et al., 2014). Briefly, each animal was anesthetized with isoflurane, decerebrated, and the neuraxis isolated in cold (5–10°C) artificial cerebrospinal fluid (aCSF) containing (in mM): 120 NaCl, 3 KCl, 1 CaCl_2_, 2 MgSO_4_, 26 NaHCO_3_, 1.25 NaH_2_PO_4_, and 20 D-glucose, equilibrated with 95% O_2_ and 5% CO_2_. The neuraxis was transected at the pontomedullary border rostrally and at the eight cervical segment caudally. The BSSC preparation was placed in a recording chamber (volume 10 mL) with ventral surface up and pinned down on Sylgard resin. The aCSF was recirculated at a perfusion rate of 12 mL min^−1^.

Medullary rhythmic slice preparations containing the preBötC were produced as described previously (Smith et al., 1991; Ruangkittisakul et al., 2006; Lorier et al., 2007; Alvares et al., 2014). Briefly, the BSSC preparation was pinned to a wax chuck, placed in the specimen vice of a vibratome (Leica VT-1000S, Concord, ON, Canada) and 100–200 μm-thick slices were sectioned serially in the rostral to caudal direction. Slices were transilluminated to identify anatomical landmarks. Structures of the subnuclei of the inferior olive were particularly useful in defining this boundary (Ruangkittisakul et al., 2006). Once at the appropriate rostro-caudal level (i.e., ~0.35 mm caudal to the caudal aspect of the facial nucleus, Smith et al., 1991; Ruangkittisakul et al., 2006; Lorier et al., 2007; Alvares et al., 2014), one rhythmic, transverse, 700 μm thick medullary slice was cut with the preBötC at the rostral surface of the slice. Slices contained the preBötC, rostral ventral respiratory group, most of the XII motor nuclei and the rostral XII nerve rootlets. Slices were pinned rostral surface up on the Sylgard resin of the recording chamber, and aCSF recirculated at a flow rate of 12 mL min^−1^. The concentration of K^+^ in the aCSF ([K^+^]_e_) was raised from 3 to 9 mM at least 30 min before the start of data collection. Slices generate rhythmic inspiratory-related activity at 3 mM [K^+^]_e_ that lasts 1–2 h (Ruangkittisakul et al., 2006). The majority of protocols in this study involved multiple interventions, and therefore required slices that produced stable inspiratory-related rhythm for extended periods. Therefore, the [K^+^]_e_ was raised from 3 to 9 mM to produce prolonged, stable rhythm (Ruangkittisakul et al., 2006).

### Nerve recording (*in vitro*)

Inspiratory-related activity was recorded via suction electrodes placed on the fourth cervical (C4) nerve rootlets of the BSSC preparations and the XII nerve rootlets of the rhythmic medullary slices. For experiments involving drug injection into the preBötC in slices, recordings were also made via a suction electrode placed directly on the rostral surface of the slice. Surface recordings were made to guide drug injections into the preBötC (Telgkamp and Ramirez, 1999). Suction electrode signals were amplified (10,000 X), filtered (300 Hz to 1 kHz), rectified and integrated. Data were acquired at 1 kHz using Axoscope 9.2 and a Digidata 1322 A/D board (Molecular Devices).

### *In vivo* preparation

Adult male Sprague-Dawley rats (250–350 g) were initially anesthetized in isofluorane (3% in 100% O_2_) and the femoral vein and artery were cannulated for drug administration, recording of arterial pressure and blood gas analysis. Isofluorane anesthesia was replaced with urethane (1.5–1.7 g/kg), which was gradually delivered intravenously. Additional doses of urethane were given to maintain anesthesia as necessary. Once on urethane, the trachea was cannulated, and the vagus nerves were resected bilaterally at the mid-cervical level to eliminate confounding effects induced by vagal reflex stimulation. The animal was then positioned in a stereotaxic frame in prone position, where the body temperature was maintained at 37°C with a servo-controlled heating pad (Harvard Apparatus). Animals were mechanically-ventilated (Harvard Apparatus Rodent Respirator Model 681) with a gas mixture of 25% O_2_, balance N_2_ (1 L min^−1^, 60 breaths per minute), and paralyzed with gallamine triethoiodide i.v. (10 mg/kg) administered intravenously. Once paralyzed, the brachial plexus was exposed dorsolaterally behind the right shoulder blade. The phrenic nerve was isolated, cut distally, placed on a bipolar platinum wire electrode and fixed in place with Kwik-Sil adhesive (World Precision Instruments, Sarasota, FL).

End-tidal O_2_ and CO_2_ were monitored from a port on the tracheal tube using a PowerLab gas analyzer (ML206, AD Instruments) to ensure that end-tidal CO_2_ remained constant throughout the experiments. Blood gases were also taken before and during the hypoxic challenges (at the fourth minute) to ensure constant pCO_2_ and to ensure comparable hypoxic stimuli were administered to control and test groups.

Phrenic nerve signals were amplified and filtered using a differential AC amplifier (model 1700, AM-systems, Sequim WA) and sampled at 2 kHz, rectified and integrated using a PowerLab 16/30 data acquisition system (AD Instruments Inc.).

### Drugs and their application

Aminooxyacetic acid, (AOAA; CBS inhibitor), bicuculline (GABA_A_ receptor antagonist) and DL-Homocysteic acid (DLH, NMDA receptor agonist) were obtained from Sigma-Aldrich. Drugs (AOAA: 0.1, 0.5, 1 mM and bicuculline: 3 μM) were dissolved in standard aCSF for BSSC preparations and in 9 mM K^+^ aCSF for rhythmic slices. For the *in vivo* experiments, DLH (10 mM) was dissolved in HEPES-buffered solution. AOAA (1 mM) was dissolved in HEPES-buffered solution containing fluorescent microspheres (1:200, 0.1 μm, yellow-green 2% solids, Life Technologies) to allow postmortem histological identification of injection sites.

In the rhythmic slice preparations, drug injection within the preBötC was established as described previously (Alvares et al., 2014). Briefly, we first used the location of the ventral respiratory column surface with a suction electrode as an approximate reference in the transverse plane to the region of most intense respiratory-related activity. The response to SP (1 μM, 10 s) at this site was recorded. The drug pipette was then systematically moved in the dorsoventral and mediolateral directions until SP evoked a frequency increase that occurred within the first breath following drug onset and was at least 2-fold greater than baseline (see **Figure 3A**). Consecutive Substance P injections were at 15 min intervals (Lorier et al., 2007; Huxtable et al., 2010). Once the preBötC was located, AOAA (0.1 and 1 mM) was microinjected and the effects recorded.

As described previously (Gray et al., 2001), drug injection into the preBötC *in vivo* was established by first tilting the head in the stereotaxic frame such that bregma was 5 mm below lambda. The preBötC was first targeted stereotaxically. A sharp glass pipette (40 μm O.D.) was placed at the following coordinates relative to the obex (in mm); 0.9 rostral, 2.0 lateral and 2.8 ventral and DLH (10 mM) was pressure injected. The preBötC was functionally identified based on the stereotypical response to local DLH that comprises a rapid-onset increase in inspiratory frequency and decrease in burst amplitude (Monnier et al., 2003). If the initial site did not produce this response, the pipette was moved, usually in the rostrocaudal plane until the expected response was observed. In the majority of cases (>80%) the expected response was observed on the first injection. The hypoxia protocols and AOAA injections were then carried out as described below. At the end of the experiment, each animal was transcardially perfused with 4% paraformaldehyde, the brainstem removed and postfixed overnight in 4% paraformaldehyde and sectioned into 50 μm slices using a vibratome (VT 1000S, Leica). For visualization of NK1 receptor expression sections were then exposed to PBS (phosphate-buffered saline) containing 10% NDS (normal donkey serum) (Sigma-Aldrich, St. Louis, MO) and 0.3% Triton X-100 (EMD Millipore, St. Louis, MO) for 1 h to reduce non-specific staining and increase antibody efficacy. Following blocking, sections were exposed overnight to rabbit anti-NK1 receptor primary antibody (1:1000; cat#AB-5060, Millipore, Billerica, MA) diluted in 1% NDS and 0.3% Triton in PBS. The next day, following wash with PBS, sections were incubated with cy3 conjugated donkey anti rabbit secondary antibodies (1:200, cat# 711-165-152, Jackson ImmunoResearch, West Grove PA) diluted in 1% NDS and PBS for 2 h, shielded from light. Sections were then washed with PBS, mounted and coverslipped with Fluorsave mounting medium (Calbiochem, Billerica, MA, USA). Finally, they were examined under a fluorescence microscope (DM5500, Leica, Nussloch, Germany) and a Hamamatsu digital camera to identify injection sites based on location of fluorescent microspheres. Injections were localized to the preBötC based on local anatomical landmarks and NK1 receptor immunolabeling. Sites were caudal to caudal boundary of the compact nucleus ambiguus, ~800 μm caudal to the caudal end of the facial nucleus at the level of the ventral respiratory column that showed the most intense NK1 receptor immunolabeling (**Figure 6**).

### Data analysis

Rectified, integrated recordings of C4, XII and phrenic nerve activities were analyzed using Clampfit (v9.2, Molecular Devices, Sunnyvale, CA) (for the *in vitro* data) and LabChart (AD Instruments, Sydney, Australia) (for the *in vivo* data). Peak detection was performed to generate burst frequency and amplitude values. For each experiment, values were normalized relative to control (pre-drug or pre-stimulus) levels, and expressed as mean ± standard error of the mean (SEM). Statistical comparison of means was performed using a one-way or two-way repeated measures ANOVA followed by the Tukey post-test (Systat Software, Inc. SigmaPlot 11.0 for Windows). Values of *p* < 0.05 were assumed significant. Group data are presented as box plots in which: the center line shows the median; box limits indicate the 25th and 75th percentiles; whiskers extend to minimum and maximum values, and; crosses represent sample means.

## Results

### Modulation of *in vitro* inspiratory burst activity by endogenous H_2_S

**Figure 1:**
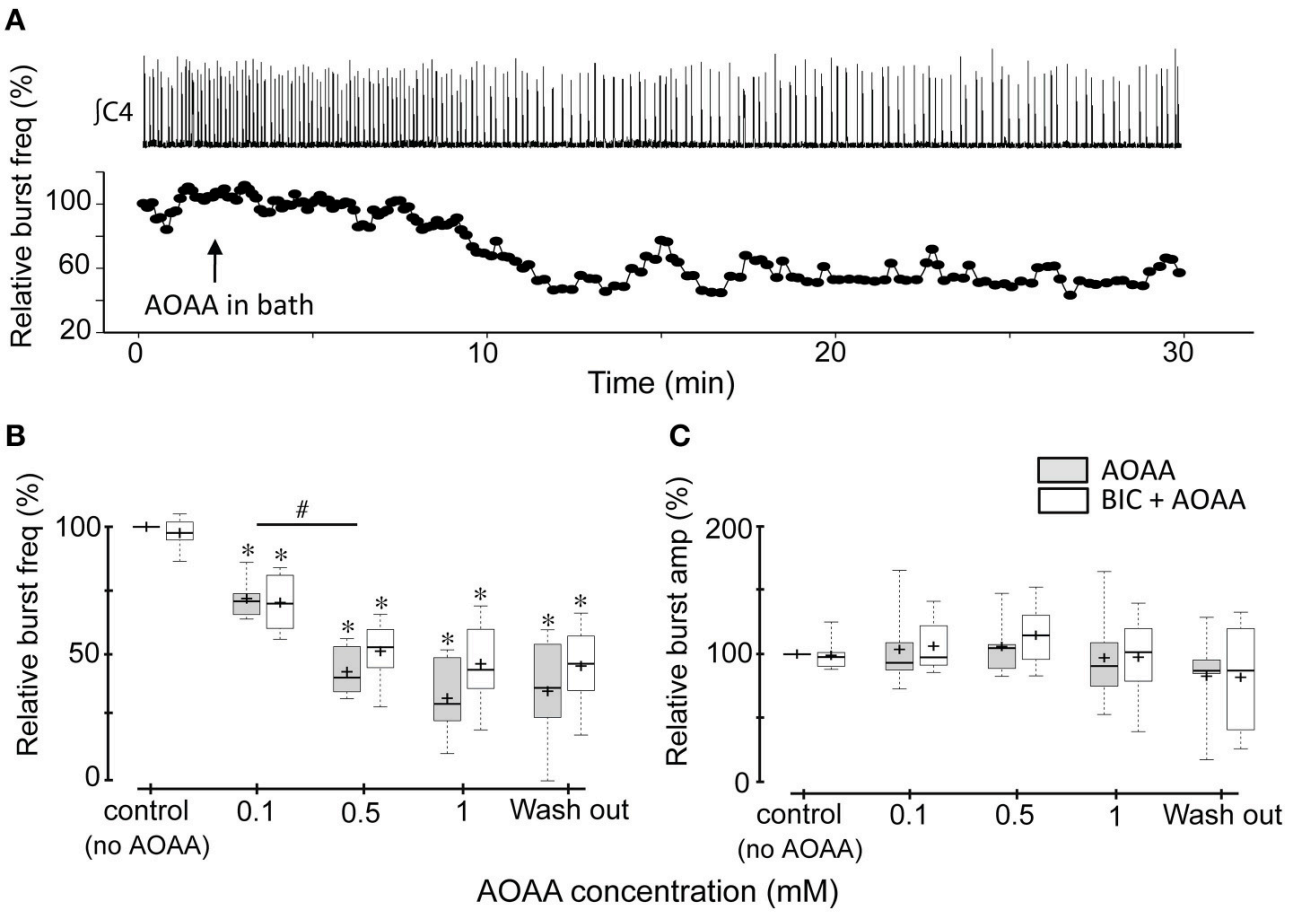
Effects of AOAA on rhythmic inspiratory-related activity recorded from the C4 ventral root of BSSC preparations. **(A)** Representative BSSC preparation showing the time course of how instantaneous frequency, calculated from the recording shown (∫C4, top trace), changes following bath application of 1 mM AOAA (indicated by the black arrow). The time scale of the nerve recordings and instantaneous frequency plots are matched. **(B,C)** Box plots of group data showing the dose-dependent effects on C4 inspiratory burst frequency **(B)** and amplitude **(C)** of AOAA bath-applied alone (*n* = 7), and, in a different group of BSSC preparations, in combination with bicuculline (3 μM, *n* = 8). The effect of bicuculline alone is presented in the control group data set. ^*^Indicates significant difference (*p* < 0.05) compared to control (baseline), #Indicates significant difference (*p* < 0.05) between the indicated doses.

Exogenous H_2_S applied via donors is reported to inhibit inspiratory rhythm *in vitro* through actions in the RTN/pFRG and excite inspiratory rhythm in the preBötC. Activation of both sites simultaneously in medullary slab preparations containing both the RTN/pFRG and preBötC results in a biphasic response to H_2_S (Hu et al., 2008; Chen et al., 2013a,b). To test the hypothesis that endogenous H_2_S modulates inspiratory activity *in vitro*, and that the net effect of H_2_S results from an interaction between differential actions in the preBötC and RTN/pFRG, we compared the effects on baseline inspiratory frequency and amplitude in BSSC (which contains the preBötC and the RTN/pFRG) and rhythmic slice (which contains the preBötC only) preparations of inhibiting H_2_S production via bath-application of incrementing concentrations of the CBS inhibitor, AOAA. AOAA was increased at 30 min intervals from control (0) to 0.1, 0.5, and 1 mM. The response of a single BSSC to bath application of 1 mM AOAA is depicted in Figure 1A. Burst amplitude did not change but inspiratory frequency decreased gradually as the AOAA washed in. The single BSSC preparation (Figure 1A) and group data (Figures 1B,C; *n* = 7) show that bath-applied AOAA had no significant effect on burst amplitude at any concentration, but caused a significant, dose-dependent decrease in inspiratory frequency. The mean relative burst frequencies were 71.7 ± 3.3% (*p* < 0.001); 42.9 ± 3.9% (*p* < 0.001) and 32.5 ± 6.2% (*p* < 0.001) of control at 0.1, 0.5, and 1.0 mM AOAA, respectively (Figure 1B).

**Figure 2:**
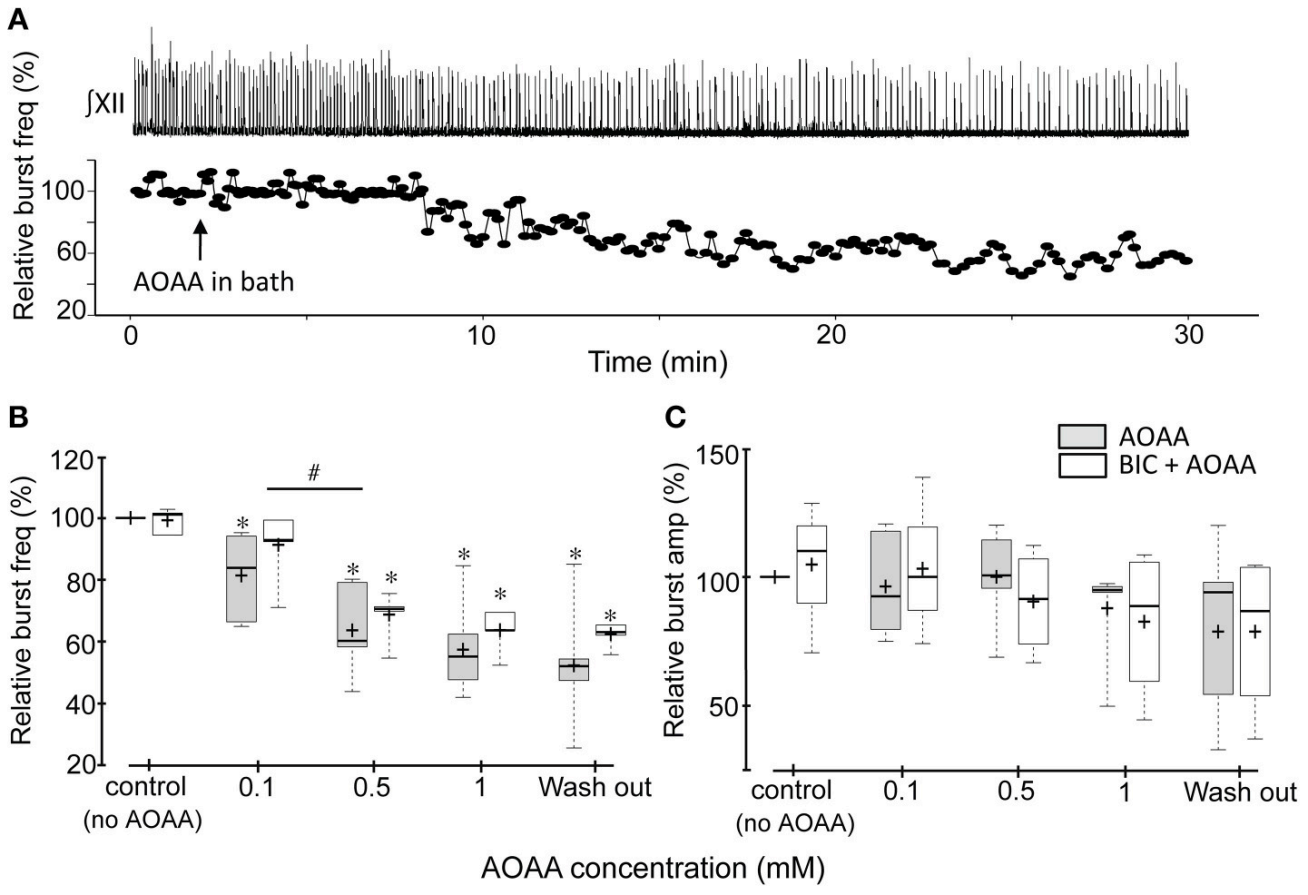
Effects of AOAA on rhythmic inspiratory-related activity recorded from the XII nerve root of medullary slice preparations. **(A)** Representative medullary slice preparation showing the time course of how instantaneous frequency, calculated from the recordings shown (∫XII nerve, top trace), changes following bath application of 1 mM AOAA (indicated by the black arrow). The time scale of the nerve recordings and instantaneous frequency plots are matched. **(B,C)** Box plots of group data showing the dose-dependent effects of bath-applied AOAA on the XII inspiratory burst frequency **(B)** and amplitude **(C)** of AOAA bath-applied alone (*n* = 7), and, in a different group of slice preparations, in combination with bicuculline (3 μM, *n* = 7). The effect of bicuculline alone is presented in the control group data set. ^*^indicates significant difference (*p* < 0.05) compared to control (baseline), #indicates significant difference (*p* < 0.05) between the indicated doses.

AOAA had similar effects on the activity of the rhythmic medullary slices (Figure 2). The single slice shown in Figure 2A responded to 1 mM AOAA with a gradual decrease in burst frequency while burst amplitude was unaffected. Group data (*n* = 7) confirm that AOAA had no effect on burst amplitude (Figure 2C), but caused a significant dose-dependent decrease in relative frequency to 81.3 ± 5.3 (*p* = 0.047), 63.6 ± 5.3 (*p* < 0.001), 57.3 ± 5.4% (*p* < 0.001) of control at 0.1, 0.5, and 1.0 mM AOAA, respectively (Figure 2B).

To exclude the possibility that the actions of AOAA on inspiratory network activity were due to potential off-target potentiation of GABAergic transmission (Wallach, 1961; Bell and Anderson, 1974; Ayala-Grosso and Urbina-Paez, 1999; Whiteman et al., 2011), the above experiments were repeated in the presence of the GABA receptor antagonist, bicuculline (3 μM)(Ren and Greer, 2006). Bicuculline on its own caused small increases in tonic activity in some preparations, but had no significant effect on baseline burst frequency or amplitude in either the BSSC (Figures 1B,C; control data) or medullary slice preparations (Figures 2B,C; control data), consistent with previous reports (Ren and Greer, 2006). AOAA effects on inspiratory frequency and burst amplitude were also not affected by bicuculline. In the BSSC preparations BIC+AOAA (Figures 1B,C; *n* = 8) had no effect on burst amplitude, but again caused a significant dose-dependent decrease in relative frequency to 70.1 ± 4.1 (*p* < 0.001); 51.2 ± 4.2 (*p* < 0.001); and 46.1 ± 5.6% (*p* < 0.001) of control at 0.1, 0.5, and 1.0 mM AOAA, respectively. Similarly, bicuculline did not alter the effect of AOAA on rhythmic slices (Figures 2B,C; *n* = 7). Compared to control, burst amplitude was unaffected and frequency was 91.2 ± 4.2% (*p* = 0.149), 68.7 ± 2.9% (*p* < 0.001) and 63.6 ± 2.5% (*p* < 0.001) of control at 0.1, 0.5, and 1.0 mM AOAA, respectively.

### Modulation of PreBötC network activity *in vitro* by endogenous H_2_S

**Figure 3:**
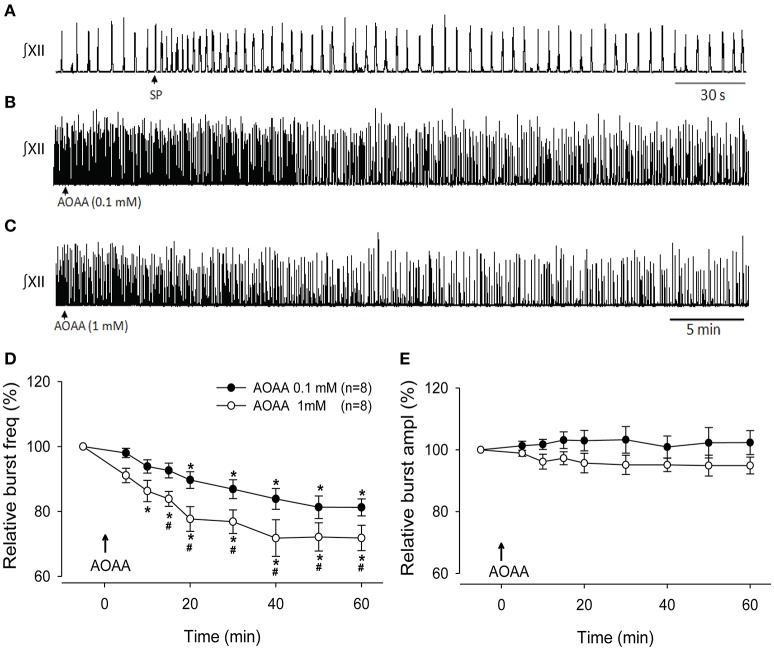
Effect of local injection of AOAA into the preBötC on activity of the rhythmic medullary slice. **(A)** XII nerve recording depicting the rapid, >2-fold, SP-evoked (1 μM, 10 s) frequency increase that is used to functionally identify the preBötC. Arrow indicates the time of SP injection. **(B,C)** XII nerve recording showing the effects on inspiratory-related activity of locally injecting AOAA (0.1 mM, 30 s), **(B)** 1 mM, 30 s, **(C)** into the PreBötC. Arrows indicate the time of AOAA injection; time scale is the same in **(B)** and **(C)**. **(D,E)** Group data (*n* = 8) showing the time course of changes in burst frequency **(D)** and amplitude **(E)** evoked by local injection AOAA into the preBötC. Arrows indicate the time of injection; time scale is the same in **(B)** and **(C)**. ^*^indicates time point when frequency first fell significantly below baseline; #indicates significant difference between values at 0.1 and 1.0 mM AOAA (*p* < 0.05).

The effects of bath-applied AOAA on inspiratory network activity could reflect actions anywhere within the BSSC or slice. To test whether endogenous H_2_S directly modulates the preBötC inspiratory network, we assessed the effects on inspiratory burst amplitude and frequency of locally microinjecting the CBS inhibitor, AOAA (0.1 and 1 mM, 30 s), into the preBötC of the rhythmic slice. Figure 3A shows the typical response evoked by SP to physiologically identify the preBötC, while Figures 3B,C show the time course of the response of a representative slice to local application of AOAA into the preBötC. Like the single slice, group time course data indicate that while burst amplitude was unaffected (Figure 3E), burst frequency decreased gradually over 30 min following local injection of AOAA and remained low for the remaining recording period (Figures 3B–D). AOAA (0.1 and 1 mM) produced a dose-dependent decrease in relative burst frequency (Figure 3D). This decrease in frequency became significantly different from control after 20 min in 0.1 mM (*p* = 0.002, *n* = 8) and 10 min in 1 mM AOAA (*p* = 0.002; *n* = 8). The inhibition reached maximum at 50 and 40 min, respectively, when frequency was 81.3 ± 3.4% (at 50 min) and 71.8 ± 5.6% (at 40 min) of baseline. The decrease in burst frequency evoked by 1 mM AOAA was significantly greater than the inhibition evoked by 0.1 mM AOAA (Figure 3D) from 15 min post-injection through to the end of the 1 h recording period (*p* = 0.049, *p* = 0.009, *p* = 0.027, *p* = 0.008, *p* = 0.042, *p* = 0.037; at minutes 15, 20, 30, 40, 50, and 60, respectively).

### Modulation of PreBötC network activity *in vivo* by endogenous H_2_S

**Figure 4:**
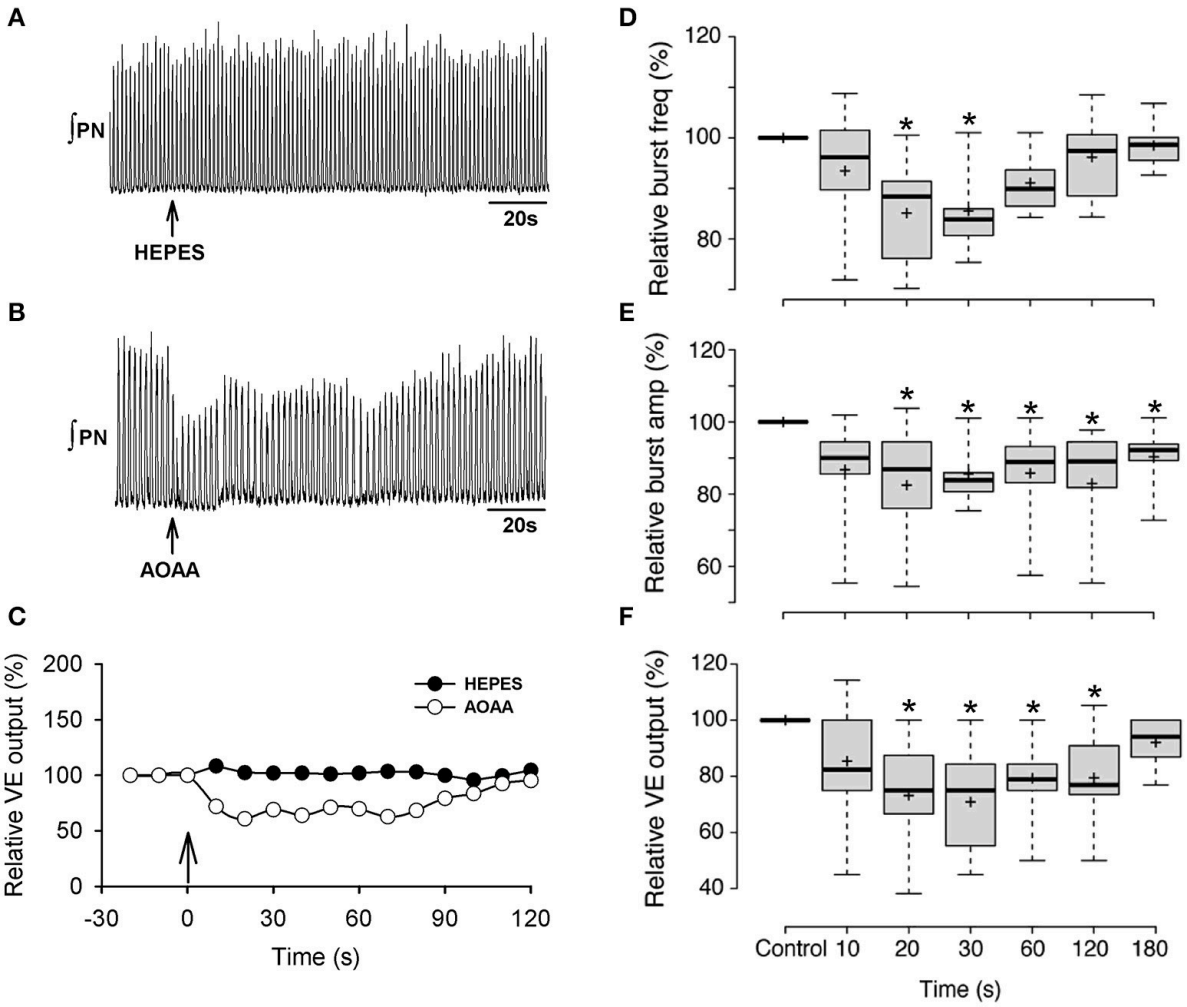
Effect on phrenic nerve activity of unilateral microinjection of AOAA into the preBötC of anesthetized, paralyzed rats breathing control gas (25% O_2_, balance N_2_). Representative recording of phrenic nerve activity showing the effects of local HEPES **(A)** or AOAA (**B**; 1 mM, 250 nL) microinjection into the preBötC on nerve output and ventilatory output for the same animal (vertical arrow in **(C)** represents onset of HEPES or AOAA injection) **(C)**. Box plots of group data showing the time course of changes in burst frequency **(D)**, burst amplitude **(E)** and ventilatory output **(F)** evoked by unilateral preBötC AOAA (1 mM, 250 nL, *n* = 10). The symbol ^*^indicates difference (*p* < 0.05) compared to baseline.

We next tested whether the preBötC network *in vivo* receives tonic modulation by endogenous H_2_S under baseline conditions. Unilateral injection of AOAA (1.0 mM, 250 nl) into the preBötC of anesthetized, paralyzed, pump-ventilated rats transiently depressed fictive inspiratory activity recorded from the phrenic nerve. Effects peaked within 20–30 s and recovered to baseline in approximately 3 min (Figure 4). Recordings of phrenic nerve activity show the time course of the responses evoked in one animal to HEPES injection (Figures 4A,C) and in another animal with the greatest sensitivity to AOAA injection (Figures 4B,C). Group data were similar. Burst frequency fell significantly below baseline reaching a nadir that was 85.5 ± 2.8% (*p* < 0.001) of control at 30 s post-injection (Figure 4D). Burst amplitude (Figure 4E) and ventilatory output (Figure 4F) also decreased to nadirs at 30 seconds post injection that were 77.5 ± 5.0% (*p* < 0.001) and 70.9 ± 6.2% (*p* < 0.001) of baseline, respectively. Frequency, amplitude and ventilatory output gradually returned to control over the next 3 min.

### Endogenous H_2_S contributes to the hypoxic ventilatory response *in vivo*

Block of the secondary hypoxic respiratory depression by application of H_2_S donors or CYS to the bath *in vitro* (Pan et al., 2010, 2011), or the cerebral ventricles *in vivo* (Li et al., 2016), suggest that exogenous H_2_S or H_2_S generated by endogenous conversion of exogenous CYS into H_2_S can both modulate the hypoxic ventilatory response. Whether H_2_S contributes physiologically to the HVR, however, is not clear. First, while H_2_S donors and CYS affected the hypoxic response *in vitro*, an inhibitor of endogenous H_2_S production did not (Pan et al., 2011). Second, the relevance of the hypoxic ventilatory response *in vitro* to the homeostatic hypoxic ventilatory response *in vivo* is unclear (Funk and Greer, 2013). Finally, ventricular application of H_2_S donors or CYS will not reproduce the spatiotemporal pattern of H_2_S that is produced in the brain by hypoxia (Li et al., 2016). A potential consequence is that manipulation of large brain regions via ventricular application of drugs could obscure endogenous actions of H_2_S if it has competing actions in different brain regions (Chen et al., 2013b). To address these limitations and test whether modulation of the preBötC network by endogenous H_2_S *in vivo* plays a physiological role in the hypoxic ventilatory response, we compared the responses of phrenic nerve activity recorded from anesthetized, paralyzed, pump-ventilated rats exposed to hypoxia first in control conditions and then again 1 h later in a second hypoxia trial that was initiated 3 min after unilateral injection of AOAA (1 mM, 250 nl) into the preBötC. Phrenic activity was recorded for 5 min of baseline (25% O_2_, balance N_2_), 5 min of hypoxia (10% O_2_, balance N_2_) and 5 min of recovery (25% O_2_, balance N_2_). Note that the effects of AOAA on baseline phrenic nerve activity shown in Figure 4 were obtained from these injections delivered 3 min prior to the hypoxia trials.

**Figure 5:**
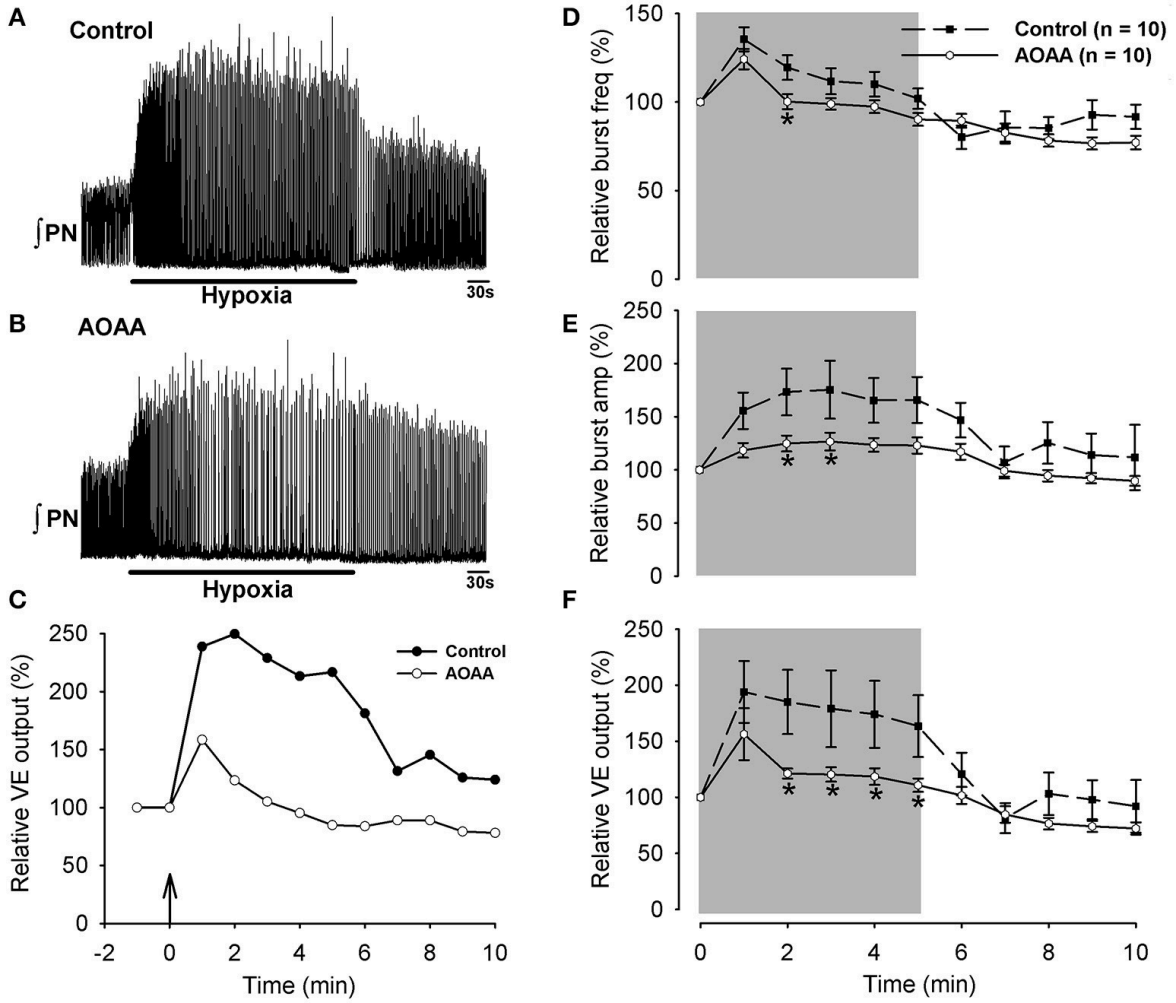
Representative recordings of phrenic nerve activity showing the response of an anesthetized rat to hypoxia (10% O_2_, Balance N_2_, for 5 min) in control **(A)** and again one h later after unilateral injection of AOAA into the preBötC **(B)**. The time course of ventilatory output calculated from the corresponding control **(A)** and AOAA traces **(B)** are shown in **(C)**. Black arrow indicates the beginning of hypoxia **(C)**. Group data showing the time course of the ventilatory response to hypoxia in control trials (*n* = 10) and after injection of AOAA (1 mM, 250 nL, *n* = 10) into the preBötC. Inspiratory frequency **(D)**, burst amplitude **(E)** and ventilatory output **(F)** are shown. ^*^indicates difference (*p* < 0.05) compared to control.

Phrenic nerve responses to hypoxia are shown for a representative animal (with the largest burst amplitude response) in control (Figure 5A) and after local injection of AOAA (Figure 5B) into the preBötC (drug injection was 3 min prior to the presentation of hypoxic gas). The kinetics of the ventilatory response of the same rat are shown in the left panels of Figures 5A–C. Group data (Figures 5D–F) indicate that in the control trials animals responded to hypoxia with the well-characterized biphasic hypoxic ventilatory response. This comprised a rapid, significant increase in inspiratory frequency, burst amplitude and ventilatory output in the first minute that were 35.3 ± 6.7%, 56 ± 17%, and 94 ± 28% greater than control. This initial increase was followed over the next 4 min by a secondary hypoxic respiratory depression, during which burst amplitude remained elevated but frequency and ventilatory output fell back toward baseline levels.

AOAA injection into the preBötC 3 min prior to hypoxia had no significant effect on the initial hypoxia-induced increase in frequency, burst amplitude or ventilatory output that occurred in the first min post hypoxia (Figures 5D–F). However, by the second minute of hypoxia, inspiratory burst frequency, burst amplitude and ventilatory output were all significantly depressed compared to the control trial. Ventilatory output remained significantly depressed compared to the control trial throughout the remainder of the hypoxic exposure. In other words, excitatory actions of the CBS-H_2_S signaling system attenuated the magnitude of the secondary hypoxic respiratory depression.

To ensure that animals experienced similar levels of hypoxia during control and AOAA trials, arterial blood gases (PCO_2_ and PO_2_), pH and hematocrit were measured during control and hypoxia exposure periods for the control trials and AOAA trials (Table 1). Values indicate that PCO_2_ and pH were well-controlled during the hypoxia treatments, that hematocrit did not change and that the hypoxia was similar during control and AOAA trials.

**Table 1:** Values of arterial pH, pCO_2_, pO_2_ and hematocrit of rats in the control and AOAA groups taken under room air and hypoxia exposure.

	**Control Group**		**AOAA Group**	
	**Baseline**	**Hypoxia**	**Baseline**	**Hypoxia**
pH	7.33 ± 0.01	7.32 ± 0.01	7.32 ± 0.01	7.32 ± 0.02
pCO_2_ mmHg	40.6 ± 1.6	39.8 ± 1.2	40.6 ± 1.0	40.6 ± 0.7
pO_2_ mmHg	146.2 ± 6.4	51.1 ± 2.3^*^	143.5 ± 6.8	56.8 ± 3.0^*^
Hct %	38.6 ± 1.3	38.2 ± 1.7	38.0 ± 1.3	37.0 ± 1.0

* *Comparison between baseline and hypoxia, p < 0.05*.

**Figure 6:**
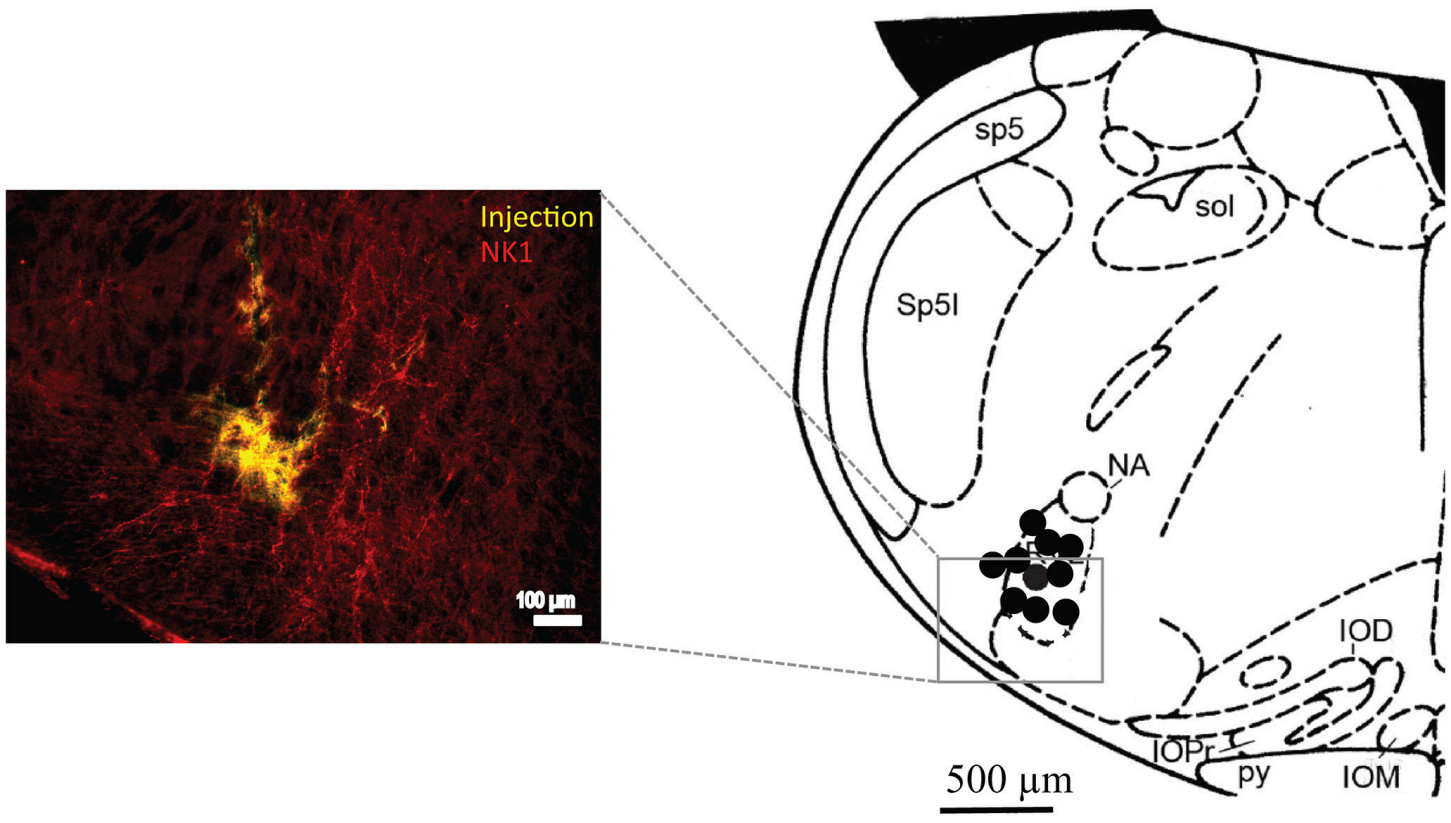
Immunohistochemical analysis of brainstem tissue sections confirm injection sites within the preBötC. Left side, a fluorescent image showing an injection site in the preBötC surrounded by red NK1 receptor immunofluorescence. The right side shows a schematic of a coronal section of the brainstem at the level of the preBötC showing sites of the AOAA microinjections (black dots). sol, solitary tract; sp5, spinal trigeminal tract; Sp5l, spinal trigeminal nucleus, interpolar part; NA, nucleus ambiguus; IOD, inferior olive, dorsal nucleus; IOM, inferior olive, medial nucleus; IOPr, inferior olive, principal nucleus; py, pyramidal tract.

Figure 6 shows a schematic of a transverse medullary hemisection taken at the rostrocaudal level of the drug injections, which corresponds to the rostrocaudal level of the preBötC. Each dot represents the location of the fluorescent dye spot used to mark the site of drug injection. Histological examination of brain sections revealed that the AOAA injections sites were within the approximate boundaries of the preBötC. Injection sites were ventral and caudal to compact division of the nucleus ambiguus, ventral to the semi compact division of nucleus ambiguus, at the level of the lateral loop of the principal nucleus of the inferior olivary nucleus and ~800 μm caudal to the caudal margin of the facial nucleus. Injections sites were also located at the rostrocaudal level of the ventral respiratory column where NK1 receptor immunolabeling appeared most intense, which is an established marker of the preBötC (Gray et al., 2001; Guyenet and Wang, 2001; Guyenet et al., 2002).

## Discussion

The role of H_2_S in central respiratory control has primarily been explored through application of H_2_S donors, H_2_S precursors and inhibitors of H_2_S synthesis to large, unspecified regions of the CNS *in vitro* and *in vivo*. However, the observation that manipulation of H_2_S signaling in the preBötC, BötC and RTN/pFRG differentially affects respiratory activity (Chen et al., 2013a,b; Donatti et al., 2014) indicates that more targeted approaches are required to unravel the modulatory control of the respiratory network by H_2_S. Thus, the objective of this study was to assess whether H_2_S signaling has a physiological role in modulating the preBötC inspiratory rhythm generating network under baseline and hypoxic conditions. Our data revealed that inhibition of endogenous H_2_S synthesis using the CBS inhibitor, AOAA, depressed baseline inspiratory rhythm recorded from BSSC and rhythmic medullary slice preparations, whether AOAA was bath-applied or injected specifically within the preBötC. Similarly, local injection of AOAA into the preBötC of anesthetized, paralyzed rats *in vivo* reduced baseline inspiratory activity and increased the secondary hypoxic respiratory depression. These data make three important contributions by showing that: (i) the preBötC inspiratory network is sensitive to modulation by endogenous H_2_S; (ii) under the baseline conditions tested, the preBötC network is tonically modulated by an endogenous, excitatory H_2_S drive; and, (iii) an endogenous, H_2_S-mediated excitation of the preBötC attenuates the secondary hypoxic respiratory depression. Important questions remain, including identification of the factors that control endogenous levels of H_2_S in the preBötC under baseline conditions and hypoxia. CBS activity is controlled by several regulatory domains that bind, for example, pyridoxal-5′-phosphate (PLP), S-adenosyl-L-methionine (SAM), heme, and Ca^2+^/calmodulin (Eto and Kimura, 2002; Wang, 2012). However, whether any of these regulators, or others, are relevant in the physiological modulation of preBötC inspiratory activity by H_2_S remains to be established.

### Limitations

We used AOAA to manipulate H_2_S in this study rather than H_2_S donors or metabolic substrates (CYS) because our objective was to assess how H_2_S contributes to the endogenous, physiological modulation of preBötC activity. Blocking CBS activity will result in gradual decrease in the level of endogenous H_2_S activity in those regions where it is produced endogenously. CYS and especially H_2_S donors have the potential of producing non-physiological concentrations of H_2_S in all brain regions including those that are not under the influence of endogenous H_2_S. We did not measure H_2_S levels pre- and post AOAA application because this is very challenging with local application protocols, but AOAA-mediated reductions in H_2_S levels have been established (Abe and Kimura, 1996; Asimakopoulou et al., 2013; da Silva et al., 2014; Kwiatkoski et al., 2014).

We selected AOAA because it is the most potent tool available to inhibit CBS-H_2_S synthesis (Asimakopoulou et al., 2013). Three of the four main limitations identified with using AOAA to assess H_2_S signaling were either not an issue in our study or were addressed directly. First, AOAA can inhibit CSE activity (Asimakopoulou et al., 2013). However, the distributions of these enzymes are tissue-specific and CSE is predominantly present in peripheral tissues. Even if AOAA acted on CSE in the CNS, this was not an issue for us because any inhibition of CSE would only lead to further decreases in H_2_S activity, which was our objective (to reduce H_2_S levels). A related issue is that H_2_S is produced in the CNS by two enzymes, CBS and 3MST. AOAA only inhibits CBS activity. Thus, our data are more likely to underestimate than overestimate the role of H_2_S. Second, AOAA can cause neuronal damage but only at concentrations much higher than those used here, and at time points of 3–6 h post injection, which are much longer than relevant in our study (Du et al., 1998). Third, higher concentrations of AOAA than used here injected intraperitoneally can increase GABA levels in the CNS after ~2 h (Wallach, 1961; Bell and Anderson, 1974; Grimm et al., 1975; Whiteman et al., 2011). To ensure that the inhibitory actions of AOAA on respiratory network activity were not due to GABAergic mechanisms, we repeated our *in vitro* AOAA dose-response experiments in the presence of bicuculline and found no evidence of AOAA-mediated potentiation of GABA actions. However, these *in vitro* experiments were not performed in hypoxia. GABA levels in brain tissue rise significantly during hypoxia, thus it will be important to test whether AOAA effects in hypoxia *in vivo* have a GABAergic component.

The final caveat with AOAA that is more difficult to control for experimentally is its potential inhibition of transaminases, including those with roles in glutamate-glutamine metabolism in astrocytes that could reduce the level of alpha ketoglutarate entering the tricarboxylic acid (TCA) cycle and compromise energy production. However, the degree to which this actually impacts energy status (in neurons or astrocytes) is controversial as there are two pathways through which the formation of the intermediate alpha-ketoglutarate (from glutamate) can enter the TCA cycle, a transamination process catalyzed by an AOAA-sensitive aminotransferase and oxidative deamination catalyzed by an AOAA-insensitive dehydrogenase enzyme (Schousboe et al., 1993; McKenna, 2007). Neurons and astrocytes differentially manage glutamate-glutamine metabolism (McKenna, 2007) and some studies suggest significant dependence of astrocytes on AOAA-sensitive processes (Farinelli and Nicklas, 1992). However, tracing of CO_2_ formation from ^14^C-labeled glutamate suggests that formation of alpha-ketoglutarate in astrocytes primarily occurs by the AOAA-insensitive oxidative deamination pathway. Specifically, concentrations of AOAA that almost completely stopped transamination had no affect on the production of ^14^CO_2_ (Yu et al., 1982). Nevertheless, we cannot exclude that in our studies inhibition of transaminase activity contributed to the effects of AOAA on the HVR.

Another observation of interest is that the effects of AOAA on baseline inspiratory activity *in vitro* were limited to reductions in frequency while both frequency and amplitude were reduced *in vivo*. The reasons for the different actions are not certain. It could reflect developmental differences. However, it may also reflect that XII burst amplitude, which is measured *in vitro*, and phrenic burst amplitude, which is measured *in vivo*, are differentially sensitive to AOAA. XII premotoneuron pools are located dorsally to the preBötC in the intermediate reticular formation (Koizumi et al., 2008; Revill et al., 2015) while phrenic premotoneurons are caudal to the preBötC in the rostral ventral respiratory group (Ellenberger and Feldman, 1988) so differential diffusion of AOAA under the two conditions may have contributed to variable amplitude effects.

### The PreBötc inspiratory network is sensitive to exogenous H_2_S

It is clear that the central respiratory network is sensitive to modulation by exogenous H_2_S, and that the effects vary with activation site (Chen et al., 2013a,b; Donatti et al., 2014). However, details of how H_2_S sensitivity maps to the ventral respiratory column and other respiratory-related nuclei are sparse. Our demonstration that application of AOAA into the preBötC *in vitro* reduces frequency is consistent with excitatory actions of H_2_S donors in the preBötC (Chen et al., 2013a,b). We also provide novel evidence that the preBötC of adult rats *in vivo* is excited by H_2_S. Local application of H_2_S donors more rostrally in the BötC has no effect on baseline ventilation *in vivo* (Donatti et al., 2014), while application to the RTN/pFRG *in vitro* inhibits respiratory activity (Chen et al., 2013a,b). Chen et al. (2013a) hypothesized that this differential sensitivity of the preBötC and RTN/pFRG to H_2_S, and the interaction between excitatory actions in the preBötC and inhibitory actions in the RTN/pFRG, underlie the biphasic response (initial decrease in frequency followed by an increase) evoked by H_2_S donors or CYS in rhythmically-active medullary slice/slab preparations (Hu et al., 2008; Chen et al., 2013a), or in the lateral ventricles *in vivo* (Li et al., 2014). Indeed, thick slices containing the preBötC and RTN/pFRG show a biphasic response, while thin slices lacking the RTN/pFRG respond with a frequency increase. In addition, the inhibitory component of the biphasic response to H_2_S donors in thick (preBötC, RTN/pFRG-containing) medullary slices is lost following ablation of RTN/pFRG (Chen et al., 2013a). Thus, the bulk of data suggest the preBötC network activity is excited by exogenous H_2_S.

### Tonic excitatory modulation of PreBötc inspiratory activity by endogenous H_2_S

The consistent reductions in basal inspiratory frequency evoked by AOAA under all experimental conditions employed here strongly support basal modulation of network excitability by H_2_S. Effects, however, vary between studies. Under *in vitro* conditions, inhibition of CBS activity with AOAA or hydroxylamine (NH_2_OH) reduced basal frequency in 700 (Figure 2) and 1,200 μm thick slices (Hu et al., 2008), but had no effect in 800–900 μm thick slices (Pan et al., 2011). Local injection of AOAA into the preBötC *in vivo* reduced frequency in anesthetized, paralyzed rats (Figure 4), but intraventricular delivery of AOAA *in vivo* did not affect basal respiratory activity in unanaesthetized rats (da Silva et al., 2014; Kwiatkoski et al., 2014; Sabino et al., 2016). Variable effects *in vitro* are difficult to reconcile but could reflect differences in slice architecture or efficacy of the enzyme inhibitor. Several factors may contribute to the discrepancies *in vivo*. The method of drug delivery is likely to be important. The effect of H_2_S on respiratory activity varies along the ventral respiratory column (Hu et al., 2008; Chen et al., 2013a). Delivery methods that affect large areas are more likely to activate multiple, competing mechanisms that may dampen, or cancel each other out. Thus, our demonstration that local application of AOAA into the preBötC *in vivo* reduces baseline frequency is compelling evidence of a role for endogenous H_2_S in modulating basal excitability of the preBötC inspiratory rhythm generating network. Another important consideration is that chemosensory feedback loops were opened in our studies via muscle paralysis and mechanical ventilation so that AOAA-induced changes in ventilatory drive would not affect blood gases. Intact feedback control loops in previous experiments (da Silva et al., 2014; Kwiatkoski et al., 2014; Sabino et al., 2016) could obscure basal modulation by H_2_S because AOAA would reduce H_2_S levels, causing a reduction in ventilation, increased CO_2_, reduced O_2_ and a compensatory increase in ventilation.

Increased inspiratory frequency following application of SAM, an activator of CBS, to rhythmic slices further suggests endogenous modulation by H_2_S (Hu et al., 2008). Modulation of respiratory network activity by the H_2_S precursor, CYS, *in vitro* or *in vivo* (Hu et al., 2008; Pan et al., 2010, 2011; Li et al., 2014), is often cited as evidence of physiological modulation by H_2_S. However, these data and the demonstration that the CBS inhibitors block the effects of CYS (Hu et al., 2008), indicate only that the network can be modulated by endogenously generated H_2_S. Exogenous CYS will increase or introduce H_2_S into any brain region capable of converting CYS into H_2_S and may therefore evoke non-physiological actions. AOAA will reduce H_2_S only from regions that are endogenously producing it under those specific experimental conditions. Consistent with this possibility is that the biphasic respiratory response evoked by exogenous CYS *in vitro* and *in vivo* is very similar to the response evoked by H_2_S donors, but unlike the monophasic inhibition evoked by inhibitors of H_2_S synthesis. Thus, data not only suggest that exogenous H_2_S excites the preBötC, data also suggest that endogenous H_2_S is a source of tonic excitatory drive to the preBötC.

### Endogenous H_2_S modulation of PreBötc inspiratory activity during hypoxia

As described above for basal conditions, H_2_S donors and CYS applied globally *in vitro* or *in vivo* attenuate the secondary hypoxic depression while the H_2_S synthesis inhibitor NH_2_OH does not (Pan et al., 2010, 2011; Li et al., 2016). Our examination of H_2_S signaling in hypoxia was limited to *in vivo* conditions due to concerns about the physiological relevance of the hypoxic ventilatory response *in vitro* to the homeostatic hypoxic ventilatory response *in vivo. In vitro* the biphasic response is evoked by a stimulus that differs substantially from physiological hypoxia; *in vitro* the hypoxic stimulus transitions from a control condition of extreme hyperoxia to anoxia in which cells at different depths in the slice all experience different stimuli (for full discussion see Funk and Greer, 2013). In contrast to earlier work *in vivo* where global inhibition of H_2_S synthesis did not reduce the secondary hypoxic respiratory depression (Li et al., 2016), inhibition of H_2_S synthesis specifically within the preBötC in our experiments *in vivo* greatly increased the secondary hypoxic respiratory depression. These data indicate that H_2_S can attenuate the secondary hypoxic respiratory depression and suggest that endogenous H_2_S attenuates the hypoxic depression when its actions are limited to the preBötC. Thus, the inability of H_2_S inhibition to reverse the secondary hypoxic depression when applied globally (Li et al., 2016) suggests that H_2_S has excitatory and inhibitory effects in different parts of the network. Inhibitory actions of H_2_S in other parts of the respiratory network have not been directly demonstrated in hypoxia but it is likely since H_2_S actions vary in other parts of the brain and also between normoxia, hypoxia and hypercapnia. For example, in hypothalamus of adult unanaesthetized rats, endogenous production of H_2_S attenuates the hypoxic ventilatory response (Kwiatkoski et al., 2014), while in unrestrained, spontaneously hypertensive rats, endogenous H_2_S acts centrally to enhance the ventilatory response to hypoxia (Sabino et al., 2016). It also acts in the brainstem to enhance the ventilatory response of adult Wistar rats to hypercapnia (da Silva et al., 2014).

The mechanisms underlying the excitatory actions of H_2_S on the central respiratory network under basal or hypoxic conditions are not well-understood. H_2_S has myriad actions on neuronal excitability (Kimura, 2013, 2014), but the few data relevant to respiratory control suggest that exogenous H_2_S stimulates inspiratory rhythm *in vitro* through activation of K_ATP_ channels and the adenylyl cyclase-cAMP pathway (Hu et al., 2008; Pan et al., 2010; Chen et al., 2013a).

In summary, we present data suggesting that cells in, or in the immediate vicinity of, the preBötC synthetize H_2_S that acts as a gasotransmitter to increase preBötC excitability under baseline conditions and also during hypoxia when its excitatory actions attenuate the secondary hypoxic depression of ventilation.

## Author contributions

Gd, JS: Study design, data acquisition, analysis and interpretation, drafting and manuscript revision; VR: Data acquisition and interpretation and manuscript revision; TA: Data acquisition and analysis; SP: Data acquisition and manuscript revision; LB: Study design, drafting and manuscript revision; GF: Study design, data interpretation, drafting and manuscript revision. All authors approved the final version.

### Conflict of interest statement

The authors declare that the research was conducted in the absence of any commercial or financial relationships that could be construed as a potential conflict of interest.
